# Single-Port Transvesical Vesico-Vaginal Fistula Repair: An Initial Experience

**DOI:** 10.1590/S1677-5538.IBJU.2024.0146

**Published:** 2024-05-07

**Authors:** Donato Cannoletta, Antony Pellegrino, Greta Pettenuzzo, Matteo Pacini, Ruben Calvo Sauer, Juan R. Torres-Anguiano, Luca Morgantini, Simone Crivellaro

**Affiliations:** 1 Department of Urology University of Illinois at Chicago Chicago IL USA Department of Urology, University of Illinois at Chicago, Chicago, IL, USA;; 2 Division of Oncology/Unit of Urology Urological Research Institute IRCCS Ospedale San Raffaele Milan Italy Division of Oncology/Unit of Urology, Urological Research Institute, IRCCS Ospedale San Raffaele, Milan, Italy;; 3 Vita-Salute San Raffaele University Milan Italy Vita-Salute San Raffaele University, Milan, Italy;; 4 Department of Urology University of Verona Azienda Ospedaliera Universitaria Integrata Verona Italy Department of Urology, University of Verona, Azienda Ospedaliera Universitaria Integrata, Verona, Italy;; 5 Urology Unit Department of Translational Research and New Technologies in Medicine and Surgery University of Pisa Pisa Italy Urology Unit, Department of Translational Research and New Technologies in Medicine and Surgery, University of Pisa, Pisa, Italy

## Abstract

**Introduction:**

Vesicovaginal fistula (VVF) is the most common urogenital fistula due to iatrogenic cause, primarily associated with gynecologic surgery (1). Although both conservative and surgical management may be considered, the optimal treatment is still uncertain and several studies were published using different techniques (open, laparoscopic or robotic) and approaches (extravesical, transvesical or transvaginal) (2–5). In this context, we aim to report our initial experience repairing VVF with Single-Port (SP) Transvesical (TV) access.

**Materials and Methods:**

Four patients with a diagnosis of VVF underwent SP-TV VVF repair between May 2022 and December 2023. Diagnosis was confirmed by cystoscopy, cystogram and in two cases by CT Urogram. Under general anesthesia, before robotic time, patients were placed in lithotomy position and a preliminary cystoscopy was performed. Fistula was noted and a 5fr stent was placed through the fistulous tract. Two ureteral stents were placed. Then, with patient supine, a transverse suprapubic 3cm incision and 2cm cystotomy were made for SP access. First step was to mark and remove fistula tract to the vagina. The edges of the vagina and bladder were dissected in order to have a closure free of tension and to create three different layers to close: vagina, muscularis layer of the bladder and mucosal layer of the bladder. A bladder catheter was placed, and the two ureteral stents were removed at the end of procedure.

**Results:**

Mean age was 53 years old and three out of 4 patients developed VVF after gynecologic surgery. Two patients underwent VVF repair 6 and 8 months after total hysterectomy. One patient developed VVF after total hysterectomy and oophorectomy followed by radiation therapy. Last patient developed VVF after previous urological procedure. Fistula diameter was between 11 and 15mm. Operative time was 211 min, including preliminary cystoscopy, stents placement and SP-access. All patients were discharged on the same day with a bladder catheter, successfully removed between post-operative day 14-18 after negative cystogram. Only in one case a ureteral stent was left because the fistula was closed to the ureteral orifice and we reported one case of UTI twelve days after surgery, treated with outpatient antibiotics. Mean follow-up was 8 months, patients were scheduled for regular follow-up visits and no recurrence was reported. All patients have at least 3 months of post-operative follow-up.

**Conclusions:**

Our experience suggests that SP Transvesical VVF repair may be considered as a safe and feasible minimally invasive treatment for small/medium fistulae (10-15mm).

Available at: http://www.intbrazjurol.com.br/video-section/20240146_Cannoletta_et_al
